# Training and Technical Assistance to Enhance Capacity Building Between Prevention Research Centers and Their Partners

**Published:** 2011-04-15

**Authors:** Antonia J. Spadaro, Jo Anne Grunbaum, Demia S. Wright, Diane C. Green, Eduardo J. Simoes, Nicola U. Dawkins, Stephanie K. Rubel

**Affiliations:** Centers for Disease Control and Prevention; Centers for Disease Control and Prevention, Atlanta, Georgia; Centers for Disease Control and Prevention, Atlanta, Georgia; Centers for Disease Control and Prevention, Atlanta, Georgia; Centers for Disease Control and Prevention, Atlanta, Georgia; ICF Macro, Atlanta, Georgia; ICF Macro, Atlanta, Georgia

## Abstract

**Introduction:**

The Centers for Disease Control and Prevention has administered the Prevention Research Centers Program since 1986. We quantified the number and reach of training programs across all centers, determined whether the centers' outcomes varied by characteristics of the academic institution, and explored potential benefits of training and technical assistance for academic researchers and community partners. We characterized how these activities enhanced capacity building within Prevention Research Centers and the community.

**Methods:**

The program office collected quantitative information on training across all 33 centers via its Internet-based system from April through December 2007. Qualitative data were collected from April through May 2007. We selected 9 centers each for 2 separate, semistructured, telephone interviews, 1 on training and 1 on technical assistance.

**Results:**

Across 24 centers, 4,777 people were trained in 99 training programs in fiscal year 2007 (October 1, 2006-September 30, 2007). Nearly 30% of people trained were community members or agency representatives. Training and technical assistance activities provided opportunities to enhance community partners' capacity in areas such as conducting needs assessments and writing grants and to improve the centers' capacity for cultural competency.

**Conclusion:**

Both qualitative and quantitative data demonstrated that training and technical assistance activities can foster capacity building and provide a reciprocal venue to support researchers' and the community's research interests. Future evaluation could assess community and public health partners' perception of centers' training programs and technical assistance.

## Introduction

Since 1986, the Centers for Disease Control and Prevention (CDC) has administered the Prevention Research Centers (PRCs) Program with the mandate to conduct health promotion and disease prevention research, training, and other related activities. From 2004 through 2009, the program funded 33 PRCs in schools of public health and schools of medicine with a preventive medicine residency. During the past decade, the PRCs' training and technical assistance (TA) activities broadened to include community participation to increase community capacity for prevention research and foster partnerships and trust among academic, community, and public health partners.

The PRCs use a community-based participatory research (CBPR) approach to actively involve community members, organizational representatives, coalitions (1,2), and researchers throughout the research process (3). This approach emphasizes training, TA, and mentoring (4,5) to enhance community partners' and researchers' capacity for research activities (6-9). Training programs included trainings in evidence-based public health, physical activity, survey design, and social marketing. Training programs in minority and underserved communities can help alleviate health disparities (10-13); they focus on meeting all partners' needs (14-17) versus solely meeting researchers' needs (18). Researchers also provide TA for their partners unrelated to research (3) that balances the researchers' need for community participation in the research and the community's need for information.

Other large research initiatives such as the National Science Foundation's Science and Technology Centers Program (19) and the Transdisciplinary Tobacco Use Research Center initiative include trainings for researchers and students but not communities (20,21). Initiatives that provide training for communities include CDC's National Academic Centers of Excellence on Youth Violence (ACEs) (22). One such center, the Harvard Youth Violence Prevention Center, "[teaches] community partners about evaluation and asset mapping" (23).

In this study, we quantified the number and reach of training programs across all 33 PRCs and determined whether the centers' outcomes varied by characteristics of the academic institution. We also explored how academic researchers and community partners benefited from training programs and TA and how these activities enhanced capacity building in PRC and communities.

## Methods

### Quantitative data collection and analysis

**Training programs and intended audience**

The PRC Program Office's information system is a Web-based information management system used to collect national evaluation data related to the PRC Program's indicators, work plans, and progress reports. Program indicators are quantitative measures to help identify program success and areas needing improvement. Before data entry, the PRC Program Office conducted a Web-based training session for all PRCs. Data entry occurred from April through December 2007. PRC staff (most often the evaluator or administrator) entered retrospective and current data to reflect fiscal year 2007 (FY 2007) (October 1, 2006–September 30, 2007). Of the 28 PRCs with available training programs, 24 implemented 99 training programs (range, 1-15; mean, 4.1) and provided data on number of people trained; 4 PRCs did not report implementation data. Where data were missing, we could not determine whether the PRC had no data to report or whether it simply did not enter the data.

**The number and type of participants trained**

We focused on 2 outcomes related to the training data: the types of audiences for whom training programs are designed and the number of people who were trained. For each training program, the PRC identified the audience type(s) for whom that training program is intended or designed. Audience types included academic faculty or other researchers, community members, community agency or other nongovernmental (NGO) representatives, health care practioners, public health employees, and public health students. A training program can be intended for 1 or many types of audiences. A training program is supported from PRC funds or included in the PRC's portfolio of activities. It may occur only once, be recurring, or be available for ongoing distribution. An *available* training program is one developed by a PRC that may or may not have been delivered during FY 2007; an *implemented* training program was delivered during FY 2007.

We examined the association between types of trained participants and 4 independent variables characterizing PRCs that delivered trainings: funding level (the amount of total funding PRCs received), actual indirect cost rate (the proportion of funds subtracted from a grant to help cover the academic institution's operating expenses: actual indirect cost rate = 100 – direct cost/total cost), type of academic institution, and type of school. We compared the mean number of people trained by type of participant across levels of the same independent variables. We categorized both funding level and the institution's actual indirect cost rate for all 33 PRCs into approximate tertiles of low, moderate, and high. We categorized type of academic institution as public, public land-grant, or private, and type of school as public health or medical.

We used Access data tables to create datasets in SAS (SAS, Inc, Cary, North Carolina) for analysis. We calculated mean, median, range, and total number of people trained by type of participant. We conducted cross-tabulations of the number of people trained by type of participant and in total with the 4 independent variables to examine their effect. We used Pearson *χ* ^2^ to test for the association of participant type with each independent variable, and where associations were found, we examined cell *χ* ^2 ^values to determine which cells (because of differences between observed and expected frequencies) were top contributors to the overall *χ* ^2^ statistic. To compare the mean number of people trained across levels of independent variables, we used the Kruskal-Wallis test because the variable representing number of people trained was not normally distributed. We used α = .05 for all significance testing.

### Qualitative data collection and analysis

Two sets of telephone interviews provided the context for community engagement in and support for training and TA. One interview guide focused on the diversity of PRC training activities (Appendix A); training activities were defined as activities that occur within training programs or separately, such as conducting needs assessments. The other interview guide focused on the diversity of PRC technical assistance and mentoring (Appendix B). The study team specified sampling criteria that guided selection of 9 PRCs for each interview guide. Each PRC determined the most knowledgeable respondent (24).

One PRC Program Office staff member conducted telephone interviews from April through May 2007. Each interview lasted 20 to 60 minutes. Definitions provided for respondents included training (transferring knowledge, skills, and competencies) (17), TA (providing guidance, support, and expertise) (25), and mentoring (a sustained relationship between 2 people that increases the mentee's self-confidence and skills) (4). Probes helped facilitate discussion and information sharing. For example, an interview question to elicit information about identification of TA needs and goals was, "Do your community partners identify for you their TA needs and goals? If yes, how?" Probes were "needs assessment" and "request from recipient." We recorded and transcribed all interviews and used ATLAS.ti version 5.2.10 software (ATLAS.ti Scientific Software Development GmbH, Berlin, Germany) for analysis. A preliminary set of codes or *start list* included overarching categories in the interview guides and subcategories or probes (26).

The study team arranged codes hierarchically and subcodes linked to broader-level codes. The interviewer read each transcript to capture recurring themes, breadth of responses, and any subtle or infrequent patterns or themes. Two study team members independently coded 2 transcripts for each interview guide (intercoder agreement: 84% for training and 89% for TA). To designate the frequency that an idea was expressed across different interview respondents, we used these terms: a couple = 2; a few = 3; some = 4 to 5; most = 6 to 8; all = 9.

## Results

### Quantitative

Although all 33 PRCs entered data into the PRC information system, only 28 PRCs entered data related to training programs. No information was available to determine whether the other 5 PRCs had training programs; therefore, data were analyzed for the 28 PRCs that provided data. The 28 PRCs reported 138 available training programs, ranging from 1 to 15 (mean, 4.9). One-third of available training programs were designed for community members and community agency or other NGO representatives, and one-fourth were designed for public health employees (Table 1). Twenty-four PRCs implemented 99 training programs, ranging from 1 to 15 each (mean, 4.1), and they trained 4,777 participants; 20% were community agency or NGO representatives, 12% were public health employees, and 9% were public health students (Table 2).

Significant associations existed between the type of participant trained and PRC funding level (*P* < .001, Table 3), actual indirect cost rate *(P 7lt; .*001, Table 4), type of academic institution (*P* <.001, Table 5), and type of school (*P* <.001, Table 6). The mean number of participants trained did not differ by PRC funding level (*P* = .80), direct cost rate (*P* = .05), type of institution (*P* = .10), or type of school (*P* = .43).

### Qualitative

We report the most salient themes that emerged from each interview guide. Each of the 9 PRCs selected to participate in an interview provided 1 respondent for a total of 18 respondents. Respondents were  8 PRC directors, 3 associate directors, 1 community liaison, 3 research scientists, 1 principal investigator, 1 communications contact, and 1 administrator. All invited PRCs participated in the interviews.

**PRC training activities**

Most respondents reported that a combination of methods was used to identify training needs such as focus groups, surveys, needs assessments, and topics raised during community advisory board meetings. One respondent mentioned identifying training needs "through focus groups with residents, community members, and the Public Health Commission . . .".

Most respondents stated that they provided training for various community partners, including community-based organizations, coalitions, community advisory board members, public health professionals, faculty, and graduate students. One respondent noted a key part of a model being tested "at our PRC . . . is training community members who participate in a coalition or advisory group." To promote the trainings, PRCs made information available through flyers, advertising, and in response to community members' inquiries at meetings.

The PRCs provided various training programs to enhance community partners' skills and knowledge. Training on lifestyle modifications and healthy living practices was offered to community residents to improve quality of life. One respondent trained the community committee on conducting needs assessments, obtaining funding, and conducting community surveys.

Community partners' roles in developing, providing, or evaluating training varied both within and across PRCs. Partners helped develop train-the-trainer activities, conceptualize training, provide funding and space, develop and implement training curricula, recruit participants, and establish training goals and objectives. Some respondents indicated that the community implemented a training to increase or develop skills among PRC staff. The community's training for PRC staff included providing information about "culturally sensitive and culturally competent health education curricula [for] the schools," understanding the roles of staff at community organizations, and working with local communities.

Most respondents cited examples of institutional support for their training programs, including the provision of space, equipment, and staff at their institutions. However, training depended on funding resources and was not highly valued for promotion and tenure, as evidenced by being "told to do less of it [training]," having more weight placed on publications, and doing "the kind of thing that is reviewed and rated and ranked by appointment, promotion, and tenure committees."

Respondents' evaluation of training activities included informal and formal methods such as face-to-face conversations and workshop evaluations. One respondent mentioned a "more sophisticated . . . capacity assessment . . . conducted with coalition members or board members by an outsider, as well as by 1 of our senior evaluators in the PRC."

**PRC technical assistance and mentoring**

Most respondents noted that both formal and informal methods were used to identify TA needs such as assessment of health priorities and community committee members' completion of a survey. TA needs were identified informally when requests were made either verbally (telephone) or in writing (e-mail). One respondent noted, "generally, if [community partners] need things, we just provide it for them."

Most respondents said they provided TA to many partners, including people, community and coalition board members, community health advisors, nonprofit organizations, community-based organizations, and county health departments. One respondent stated that TA recipients included people "involved in health promotion [and] disease prevention in the communities that we work in . . . for example, if the health department wanted us to [provide] technical assistance on some project." TA was provided directly and indirectly by e-mail, meetings, and telephone.

The TA topics varied according to the PRCs' research and community partners' needs. Most respondents provided TA on physical activity research. In addition, TA was offered to community partners for grant writing, understanding CBPR, nutrition, and evaluation. Most respondents indicated that the community provided TA to increase or develop skills among PRC staff on such topics as disaster preparedness, effective communication with partners, and community engagement.

Most respondents reported that institutional support for their TA included providing space and equipment at their institutions. As with training, TA depended on funding resources and was not highly valued for promotion and tenure. One respondent noted that more weight was placed on publications than hiring of additional faculty and staff for TA.

Most respondents evaluated TA both informally and formally. They evaluated TA by counting additional grants obtained or community services provided, checking with the community to see how programs progressed, doing workshop evaluations, obtaining anecdotal reports from TA recipients, and evaluating change continually. One respondent mentioned that evaluation of TA did not occur at their PRC.

The PRCs also engaged in mentoring relationships with their community partners. For example, a staff member at 1 PRC had a mentoring relationship with a health commissioner regarding "developing new programs and evaluation contracts." On an organizational level, 1 respondent had a mentoring relationship with an organization that was part of the PRC's community committee. This organization works with the PRC's core research project in areas such as grant writing, evaluation, and strategic planning. One respondent gave examples of PRC staff mentoring, which included the PRC director's mentoring relationship with a school vice principal and the respondent's work with state health department staff "in chronic diseases . . . to develop new programs." Another respondent reported a 3-year mentoring relationship with an intern.

## Discussion

This study demonstrates both the reach of the PRC training programs and the community context where training, TA, and mentoring occur. During the 1-year funding cycle studied, the PRCs trained approximately 4,700 people. The qualitative data demonstrate numerous methods PRCs use to identify the training needs of their community members and other partners. In addition, the data show the extensive involvement of community partners in developing and implementing training programs.

Qualitative data demonstrate that PRCs also engage in less formal training activities such as TA and mentoring. PRCs use both formal and informal methods to determine TA needs of their partners, and TA is reciprocal between PRCs and their communities. Some PRCs have formal mentoring relationships with individual and organizational-level community partners.

Of interest are the associations between type of participant trained and PRC funding level, actual indirect cost rate, type of academic institution, and type of school. For all 4 variables, these associations may reflect that different academic institutions and schools target different audiences. On average, public land-grant institutions trained twice as many participants as private institutions and 4 times as many participants as other public institutions. These findings are consistent with the mission of public land-grant institutions, which is to support a vision for higher education including public service and outreach (27).

Study limitations include that the data reflect FY 2007 and may not represent all years of the funding cycle (FYs 2004-2009). Also, only 28 PRCs provided data on training programs and 24 provided data on the number of people trained. Each interview topic was conducted with only 9 PRCs, which limits the generalizability of findings.

Not enough information is available regarding training activities for communities funded through large research initiatives. Although the Transdisciplinary Tobacco Use Research Center provides training for researchers and students, it does not provide training for communities (21). The National Institutes of Health's (NIH's) National Center for Research Resources supports training for approximately 30,000 NIH-funded biomedical investigators across the country; however, no published data specify the number of training programs or the number and type of participants trained (28). CDC's ACE injury and violence prevention projects "connect [both] academic and community resources" (22) via training as part of their centers' activities. However, no published data exist. Thus, we cannot compare PRC training programs and recipients to other large research initiatives.

Our study has implications for researchers, community partners, and public health practitioners who engage in CBPR. Results demonstrate that training and TA can foster capacity building and provide a reciprocal venue to support researchers' and the community's research interests. However, we found that incentives for researchers to engage in training activities and TA may be jeopardized because institutional support is contingent on resources and the activities are not highly valued for promotion and tenure. Lack of support could limit faculty from providing needed training programs and TA to community partners that face staff turnover and changes in staff assignments.

Data analysis for FY 2008 and FY 2009 is under way and will help validate the data for FY 2007. Future evaluation could assess capacity change resulting from training and TA, community and public health partners' perception of PRC training programs and TA, the importance of these activities for CBPR, and how they enhance community engagement and increase community capacity (24).

## Figures and Tables

**Table 1:** Available and Implemented PRC Training Programs, by Type of Intended Audience, Fiscal Year 2007

Intended Audience Type^a^	**Available^b^,^c^ (N = 138), N (%)**	**Implemented^c^,^d^ (N = 99), N (%)**
Academic faculty or other researchers	29 (21)	17 (17)
Community members	46 (33)	39 (39)
Community agency or other nongovernmental organization representatives	44 (32)	39 (39)
Health care practitioners	31 (22)	28 (28)
Public health employees	33 (24)	27 (27)
Public health students	1 (1)	1 (1)
Other	101 (73)	76 (77)

Abbreviation: PRC, Prevention Research Center.

a Type of intended audience means for whom the training program is intended or designed.

b Twenty-eight PRCs provided data on available training programs. An available training program is one developed by a PRC that may or may not have been delivered during FY 2007.

c Percentages total >100% because each training program may be designed for multiple audience types.

d Twenty-four PRCs provided data on implemented training programs. An implemented training program is one that was delivered during FY 2007.

**Table 2:** Number of Participants^a^ Trained by PRCs, by Type of Participant, Fiscal Year 2007

**Type of Participant** b	**Range** c	**Mean** c	**Median** c	No. of People Trained(%)
Academic faculty or other researchers	1–70	18	8	349 (7)
Community members	2–85	30	27	383 (8)
Community agency or other nongovernmental organization representatives	2-575	64	13	957 (20)
Health care practitioners	1-123	26	10	307 (6)
Public health employees	2-184	39	15	550 (12)
Public health students	1-183	30	11	450 (9)
Other	1-290	42	15	890 (19)
Not specified^d^	1-419	81	12	891 (19)
**Total**				4,777 (100)

Abbreviation: PRC, Prevention Research Center.

a Reflects data from the 24 PRCs that implemented 99 training programs.

b Number of PRCs varies by type of participant.

c Range, mean, and median number of participants trained per PRC reporting that type of participant.

d Eleven PRCs did not specify type of participant, including 1 PRC that trained 419 participants.

**Table 3:** Number of Participants Trained by PRCs, by Type of Participant and by Tertile of Total PRC Funding,^a^ Fiscal Year 2007

**Type of Participant** b	Total Funding		
	<$1.3 Million, n (%)	$1.3M-2.6 Million, n (%)	>$2.6 Million, n (%)
Academic faculty or other researchers	198 (8)	59 (12)	92 (5)
Community members	217 (9)	59 (12)	107 (6)
Community agency or other nongovernmental organization representatives	623 (26)	61 (12)	273 (15)
Health care practitioners	163 (7)	11 (2)	133 (7)
Public health employees	217 (9)	39 (8)	294 (16)
Public health students	139 (6)	94 (19)	217 (12)
Other	876 (36)	179 (36)	726 (39)
**Total**	2,433 (100)	502 (100)	1,842 (100)

Abbreviation: PRC, Prevention Research Center.

a Number of PRCs by funding tertile: <$1.3 million = 10; $1.3-$2.6 million = 3; >$2.6 million = 11 (based on 24 PRCs' reporting data).

b *Χ^2^* = 294, degrees of freedom = 12, *P* < .001

**Table 4:** Number of Participants Trained by PRCs, by Type of Participant and by PRC Actual Indirect Cost Rate,^a^ Fiscal Year 2007

**Type of Participant** b	Actual Indirect Cost Rate		
	<20%, n (%)	20%-<30%, n (%)	≥30%, n (%)
Academic faculty or other researchers	196 (8)	64 (7)	89 (7)
Community members	154 (6)	135 (14)	94 (8)
Community agency or other nongovernmental organization representatives	615 (24)	205 (21)	137 (11)
Health care practitioners	104 (4)	49 (5)	154 (13)
Public health employees	212 (8)	149 (15)	189 (16)
Public health students	147 (6)	46 (5)	257 (21)
Other	1,162 (45)	331 (34)	288 (24)
**Total**	2,590	979	1,208

Abbreviation: PRC, Prevention Research Center.

a Actual indirect cost rate (the proportion of funds subtracted from a grant to help cover the academic institution's operating expenses) was categorized into approximate tertiles of low, moderate, and high indirect cost rates. Number of PRCs by actual indirect cost rate: <20% = 6; 20% to <30% = 7; ≥30% = 11 (based on 24 PRCs' reporting data).

b *Χ^2^* = 619, degrees of freedom = 12, *P* < .001.

**Table 5:** Number of Participants Trained by PRCs, by Type of Participant and by Type of Academic Institution^a^, Fiscal Year 2007

**Type of Participant** b	Type of Academic Institution		
	Public, n (%)	Public Land Grant, n (%)	Private, n (%)
Academic faculty or other researchers	151 (9)	134 (6)	64 (6)
Community members	157 (10)	188 (9)	38 (4)
Community agency or other nongovernmental organization representatives	313 (21)	641 (29)	3 (<1)
Health care practitioners	69 (5)	107 (5)	131 (12)
Public health employees	309 (20)	234 (11)	7 (1)
Public health students	69 (5)	165 (8)	216 (20)
Other	451 (30)	715 (33)	615 (57)
**Total**	1,519	2,184	1,074

a Number of Prevention Research Centers by type of academic institution: public = 14; public land grant = 5; private = 5 (based on 24 PRCs' reporting data).

b *Χ^2^* = 977, degrees of freedom = 12, *P* <.001.

**Table 6:** Number of Participants Trained by PRCs, by Type of Participant and by Type of School^a^, Fiscal Year 2007

**Type of Participant** b	School of Public Health, n (%)	School of Medicine, n (%)
Academic faculty or other researchers	224 (7)	125 (7)
Community members	265 (9)	118 (7)
Community agency or other nongovernmental organization representatives	337 (11)	620 (35)
Health care practitioners	167 (6)	140 (8)
Public health employees	315 (11)	235 (13)
Public health students	369 (12)	81 (5)
Other	1314 (44)	467 (26)
**Total**	2,991	1,786

a Number of Prevention Research Centers by type of school: public health = 17; medicine = 7 (9 PRCs did not report type and number of people trained).

b *Χ^2^* = 497, degrees of freedom = 6, *P* <.001

## Appendices

### Appendix A. Interview Guide for Diversity of PRC Training Activities

Introduction

Hello, my name is [name] with the CDC Prevention Research Centers' Program Office, Research and Evaluation team, and I'm calling for our scheduled interview. As I mentioned previously, our interview should take between 30 to 60 minutes. Is this still a good time to talk? **If not, reschedule, however, attempt to complete the interview at the designated time.**

Thank you for taking the time to talk with me about your Prevention Research Center. We, along with Macro International and the Collaborative Evaluation Design Team (the national evaluation advisory group) are collaborating on a national evaluation of the PRC program. Right now, we are conducting a series of interviews with representatives across the PRCs as part of a special study that will provide a qualitative assessment of the program.

The purpose of this interview is to increase our understanding of the diversity of training with communities and partners. These interviews will help provide that information in a comprehensive and systematic way. Your participation is critical to this effort, and we appreciate your willingness to participate in this interview.

Before we begin, I want to let you know that the interview will be taped and subsequently transcribed. Is that OK with you? I will be the only person to see the full transcript of the tape. Do you have any questions about the interview process before we begin?

First, I would like to find out about the recipients of trainings conducted by your PRC other than trainings specifically for students.

1. Other than for students, for what audiences do you conduct trainings?

2. Where are those audiences located?

Now, I would like to ask about the nature of and rationale for PRC trainings.

3. What types of trainings has your PRC conducted for your community and partners?

4. What was the purpose of the training?

5. How was the training need identified?

6. Was the training newly developed or an ongoing activity?

Now, I would like some information on the engagement of your community partners in training activities.

7. What role do community partners play in developing, providing, or evaluating training activities?

Probe (request descriptions of their roles in the following areas):

Development:

- Conceptualizing the training activity and method (eg, train-the-trainer, web-based trainings, peer-to-peer trainings, training manuals) 
- Providing or obtaining funding for the training
- Establishing training goals or objectives
- Developing or planning the training activity

Implementation:

- Conducting or providing training activities
- Providing space
- Facilitating collaboration between the center and the partnering community or other partners

Evaluation:

- Evaluation of the training activity

8. How do your community partners get involved in training activities?

Probe: Are they solicited? Do they volunteer?

Now, I would like to talk about how community and PRC capacity are enhanced through training.

9. What specific knowledge or skill-building is targeted through PRC trainings for community partners?

Probe: community assessment: identifying community needs, strengths, and assets; performing community-based participatory research; policy development: establishing goals and strategies; evaluation; or grant writing

10. Has the community implemented trainings to increase knowledge or developed skills among PRC staff?

11. **If yes to Q 10 —** How did the PRC identify its training needs and let the community know about these needs?

12. **If yes to Q 10 —** Have training efforts fostered the PRC's ability to utilize skills on an ongoing basis?

Probe: train-the-trainer; peer-to-peer training

Thank you for your responses thus far. We are in the final stage of the interview and there are only a few questions remaining. The final set of questions asks about the value of PRC training activities overall and if any of the training is tied to PRC research.

13. In what ways does your institution demonstrate its value for training?

Probe: the provision of space; additional faculty and staff; promotion and tenure policies

14. Are your PRC's training activities related to your PRC's research? If yes, please explain.

15. **If yes to Q 14** **—** Are these trainings only for PRC staff, or are there trainings related to your PRC research for community partners?

16. Is there anything else that you would like to discuss related to training activities, community and partner engagement, or institutional support for training that we did not talk about yet?

Our interview has concluded. Your participation is very much appreciated and is critical toward increasing knowledge and understanding about the diversity of training activities with communities and partners. Thank you so much for your time.

### Appendix B. Interview Guide for Diversity of PRC Technical Assistance and Mentoring

Introduction

Hello, my name is [name] with the CDC Prevention Research Centers' Program Office, Research and Evaluation team and I'm calling for our scheduled interview. As I mentioned previously, our interview should take between 30 to 60 minutes. Is this still a good time to talk? **If not, reschedule, however, attempt to complete the interview at the designated time.**

Thank you for taking the time to talk with me about your Prevention Research Center. We, along with Macro International and the Collaborative Evaluation Design Team (the national evaluation advisory group) are collaborating on a national evaluation of the PRC program. Right now, we are conducting a series of interviews with representatives across the PRCs as part of a special study that will provide a qualitative assessment of the program.

The purpose of this interview is to increase our understanding of the diversity of technical assistance with communities and partners. These interviews will help provide that information in a comprehensive and systematic way. Your participation is critical to this effort, and we appreciate your willingness to participate in this interview.

Before we begin, I want to let you know that the interview will be taped and subsequently transcribed. Is that OK with you? I will be the only person to see the full transcript of the tape. Do you have any questions about the interview process before we begin?

First, let's talk about the PRC's process of providing and evaluating technical assistance for your community partners.

1. Do your community partners identify for you their TA needs and goals? If yes, how?

Probe: needs assessment; request from recipient

2. Do you have a mechanism to track or monitor TA that you provide? If yes, what is it?

3. Do you evaluate your TA, and if so, how?

4. What PRC staff provide TA?

Next, let's talk about the recipients of TA, the mechanisms used to provide TA, and the frequency and type of TA.

5. Which community partners are the recipients of TA?

6. What mechanisms do you use to provide TA?

Probe: funded projects; consultations; e-mails; meetings; telephone conferences; published guides

7. Is the TA provided routinely or on a case-by-case basis?

8. If routinely, has this routine TA helped provide institutionalization of the topic or skill for continuation of projects and to achieve desired outcomes? If yes, please explain.

9. Are there any formal agreements in place to provide TA?

10. Do your partners know the types of TA they could receive from the PRC?

Probe: tailored; overall support

11. About how much time per week does your PRC spend providing TA to community partners?

12. About how many times per week does your PRC call on community partners for TA?

Now, I would like to talk about some of the topical areas for providing TA.

13. What are the topics or skills that you provide TA on for your community and partners?

Probe: an area of expertise; understanding community-based participatory research; public health policy development; health care delivery

14. What are the topics that your PRC receives TA on from community partners? (allow answers that PRC does not receive TA from partners)

Probe: an area of expertise; understanding community-based participatory research; public health policy development; health care delivery

Thank you for your responses thus far. We are in the final stage of the interview and there are only a couple of questions remaining. The final set of questions asks about your institution's value for TA and mentoring relationships.

15. In what ways does your institution demonstrate its value for TA?

Probe: the provision of space and communication tools; additional faculty and staff; promotion and tenure policies

16. Do you have a mentoring relationship with a community partner? By mentoring relationship, I mean a sustained relationship and partnership between 2 people, . . . in which the *more experienced person* or mentor offers encouragement and support to increase the self-confidence and skills of the *less experienced person* or mentee. **If yes, please describe it.**

17. Is there anything else that you would like to discuss related to TA activities with communities and partners that we did not talk about yet?

Our interview has concluded. Your participation is very much appreciated and is critical toward increasing knowledge and understanding about the diversity of TA activities with communities and partners. Thank you for your time.

Definitions:

Mentoring is "a sustained relationship and partnership between 2 people, one of whom is more experienced than the other in which the *more experienced person* or mentor offers encouragement and support to increase the self-confidence and skills of the *less experienced person* or mentee" (4).

Training is transferring knowledge, skills, and competencies to individuals who are in a position to use what they have learned (17).

Technical assistance (TA) provides guidance, support, and expertise to an identified group or agency as the group works toward a desired outcome (24).
